# Two Interrogated FBG Spectral Linewidth for Strain Sensing through Correlation

**DOI:** 10.3390/s17122837

**Published:** 2017-12-07

**Authors:** Shih-Hsiang Hsu, Kuo-Wei Chuang, Ci-Syu Chen, Ching-Yu Lin, Che-Chang Chang

**Affiliations:** 1 Department of Electronic Engineering, National Taiwan University of Science and Technology, Taipei 10607, Taiwan; d9780334@gmail.com (K.-W.C.); super25574585@hotmail.com (C.-S.C.); 2 School of Medical Laboratory Science and Biotechnology, College of Medical Science and Technology, Taipei Medical University, 250 Wu-Hsing Street, Taipei 11031, Taiwan; cylin@tmu.edu.tw; 3 Graduate Institute of Translational Medicine, College of Medical Science and Technology, Taipei Medical University, 250 Wu-Hsing Street, Taipei 11031, Taiwan

**Keywords:** fiber optics sensors, remote sensing and sensors, fiber Bragg gratings, spectral linewidth, correlation

## Abstract

The spectral linewidth from two cross-correlated fiber Bragg gratings (FBGs) are interrogated and characterized using a delayed self-homodyne method for fiber strain sensing. This approach employs a common higher frequency resolution instead of wavelength. A sensitivity and resolution of 166 MHz/με and 50 nε were demonstrated from 4 GHz spectral linewidth characterization on the electric spectrum analyzer. A 10 nε higher resolution can be expected through random noise analyses when the spectral linewidth from two FBG correlations is reduced to 1 GHz. Moreover, the FBG spectrum is broadened during strain and experimentally shows a 0.44 pm/με sensitivity, which is mainly caused by the photo elastic effect from the fiber grating period stretch.

## 1. Introduction

Fiber sensors offer excellent advantages in characterizing temperature, concentration, velocity, force, and strain due to their low optical insertion loss, immunity to electromagnetic interference, low cost, light weight, small footprint, and high sensitivity. In the fiber sensing fields, numerous research accomplishments including the fiber Bragg grating (FBG) [1], photonic crystal fiber (PCF) [2], and single mode fiber [3] have advanced fiber strain ε, the length change ratio to the original fiber length on the FBG [4,5,6,7,8,9,10], and sensing, especially temperature independent multi-sensing and the spatial division multiplexing system [11]. In the last decade, the FBG and associated technologies have been widely employed to obtain highly sensitive and accurate monitoring results for providing superior and intelligent security sensors. Furthermore, the FBG grating has found its way into widespread applications in both the telecommunication and sensor fields [4]. 

However, the sensing resolution is limited to the resolved wavelength accuracy on the measurement system. When the FBG strain resolution was 1 με, wavelength accuracy at the picometer level [9] was required and could only be demonstrated in a high standard optical spectrum analyzer (OSA). For higher resolution, a more expensive wave meter is necessary to indicate smaller wavelength shifts with respect to the corresponding sensing.

Recently, a 34 nε FBG resolution was enabled using dual-comb spectroscopy [5], and a 10 nε resolution was further demonstrated using a narrow linewidth tunable laser interrogated FBG with a cross-correlated wavelength resolution algorithm [6,7]. To avoid the sub-picometer wavelength precision required for FBG strain sensing [5,7], an interrogated linewidth from two correlated FBG spectra incorporated with the broadband source is proposed to gauge the strain using a self-homodyne method in the electric spectrum analyzer (ESA). Other than a cross-correlation algorithm employed to calculate the Bragg wavelength difference [7], we directly characterized the linewidth in frequency from two reflective FBG spectra for strain sensing. Our previous work was very preliminary about the fiber strain sensitivity analyses and gave a rough resolution estimate [12]. This time, two FBGs were cascaded to interrogate with the broadband source for further study and simulation on the cross-correlation. The correlation and delayed self-homodyne linewidth theorems were illustrated and simulated for the comparison between theory and experiments in the following sections. The spectral linewidth broadening for one FBG, crucial for accurate linewidth characterization, is further discussed using the grating period and effective index effects. The strain sensing Bragg wavelength shift was also tested for the photo elastic effect study. Moreover, the linewidth sensing resolution on an ESA was theoretically calculated based on the random noise analysis based on the cross-correlation algorithm. Finally, a fiber delayed self-homodyne interferometer, a high speed receiver, and an ESA were incorporated together to demonstrate high resolvable and precise bandwidths through two interrogated FBG spectra for FBG strain sensing.

## 2. Design

The main FBG optical performance measures are the resonant wavelength and linewidth. The Bragg wavelength shift sensitivity is determined by both the grating period and the effective index. When an external force is applied onto a fiber in its axial direction, stretch or compression is performed to manipulate the resonant wavelength during strain. According to the elasticity theory, the solid-state material strain must be expressed as tensors. If there is no shearing force and the external force is in the axial direction, the fiber strain causes the Bragg grating period and fiber grating mode index to be changed accordingly. The Bragg wavelength shift, ∆*λ_B_*, can then be derived as follows [13]:

(1) $$\Delta \lambda_{B}=\lambda_{B}\{1-\frac{n^{2}}{2}[1-vp_{12}-vp_{11}]\}=\lambda_{B}(1+\gamma )\lambda_{B}\varepsilon_{z}$$

where *λ_B_* is the Bragg wavelength, and *n* is the effective index of the fiber grating mode. *ν* is the Poisson’s ratio. *p*_12_ and *p*_11_ are the photo elastic coefficients of the silica fiber. *γ* is the effective elastic-optic coefficient, and *ε*_z_ is the strain in the axial direction.

On the other hand, the linewidth ∆*λ* of the FBG reflection spectrum is defined as the wavelength spacing between two half maxima on either side of the central peak and can be approximately described as follows [14]:

(2) $$\Delta \lambda =\frac{\lambda_{B}^{2}}{\pi nL}{\left(\kappa^{2}L^{2}+\pi^{2}\right)}^{1/2}$$

(3) $$\lambda_{B}=2\Lambda n$$

where *κ* is the modal coupling coefficient, *L* is the fiber grating length, and Λ is the spatial grating period.

The linewidth ∆*λ* is related to the Bragg wavelength *λ_B_*, modal coupling coefficient *κ*, the effective index of fiber grating mode *n*, and the fiber grating length *L*. After combining Equations (2) and (3), we obtain the following linewidth:

(4) $$\Delta \lambda =\frac{\lambda_{B}2\Lambda n}{\pi nL}\pi {\left(1+\frac{\kappa^{2}L^{2}}{\pi^{2}}\right)}^{1/2}.$$

From Equation (4), the linewidth ∆*λ* can be simplified to be related to the fiber grating length *L*, grating spatial period Λ, and the Bragg wavelength *λ_B_*, which depends on two main parameters—the Bragg wavelength shift ∆*λ_B_* and the effective elastic-optic coefficient *γ* from Equation (1).

Without considering of the elastic-optical coefficient, the commercial software GratingMOD from RSoft utilizes the ∆*λ_B_*, *L*, and Λ parameters to derive the FBG related linewidth. When axial strain *ε*_z_ is applied to the fiber, the waveguide length experiences a variation ratio of *ε*_z_ as the effective indices for the longitudinal direction, FBG length, and grating period. In a similar way, the waveguide width has an effect in the transverse direction as *ε*_z_ /(1 + *ε*_z_). The 8.2 μm diameter and 0.0036 index difference in the optical fiber is taken on the 10-mm-long FBG for strain analyses. When the core index is set as 1.4682 at a 1550 nm wavelength, the grating period could be assumed as 528 nm and the index modulation change could be set as 0.00017 [15] for the uniform sinusoidal variation grating. 

Correlation is used to compare the similarity between two functions and the result can reach maximum at the time when the two functions match best. Correlation can also measure the delay of a certain system. The cross-correlation between two signals *u*(*t*) and *v*(*t*) is defined as follows:

(5) $$w\left(t\right)=u\left(t\right)⋆v\left(t\right)=\int_{-\infty }^{\infty }u^{*}\left(\tau \right)v\left(\tau +t\right)d\tau .$$

In Equation (5), the complex conjugate, u*(*τ*), makes the definition work regardless of whether *u*(*t*) is real or complex-valued. There are two types of correlation, cross and auto. The cross-correlation is used to find where two similar signals match between *v*(*t*) and a delayed *u*(*t*). On the other hand, auto-correlation is used to characterize two identical signals. 

Two FBGs’ reflective spectra generated from GratingMOD through strain sensing are treated as the cross-correlation, and its calculation demonstrates 1.55 pm/με at a 1550 nm wavelength, which is the same as the Bragg resonance wavelength shift without considering the effective elastic-optic coefficient *γ*. The photo elastic effect could be derived from comparing the theoretical data and experimental characterization on the linewidth and Bragg wavelength shift. 

## 3. Experiments and Results

**Figure 1:**
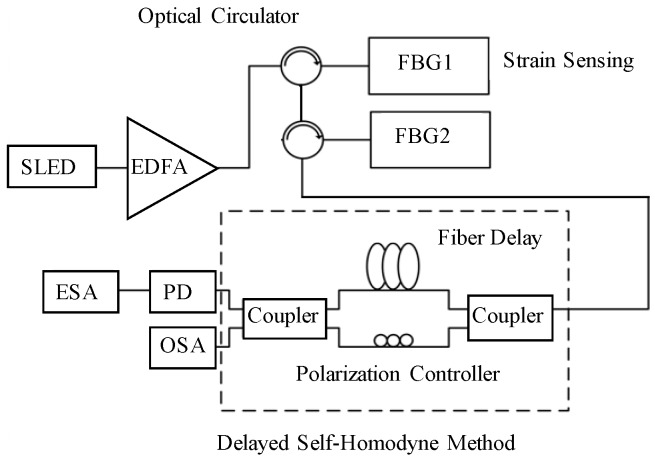
The fiber strain testing setup for two reflective FBG spectral linewidth characterization.

The output from a 1550 nm superluminescent light emitting diode (SLED) from Amonics (ASLD155-200-B-FC), boosted by the erbium-doped optical amplifier (EDFA), is directly injected into the first FBG (FBG1) through an optical circulator. The FBG1 reflection spectrum coupled with the FBG2 signal goes through another optical circulator. The reflective spectra from two FBGs are correlated for analysis through the delayed self-homodyne-based interferometer, as shown in Figure 1. This system uses two couplers to involve two different arm lengths to form the Mach–Zehnder interferometer (MZI). The optical path difference in MZI requires a longer path than the SLED coherence length, and the polarization controller is installed in one of two arms to obtain the highest output power. The two output ports are immediately followed and connected to the OSA and photodetector (PD), respectively, for the optical output characterized using the OSA (HP 70951B) and the electrical output delivered to a 40 Gb/s photodetector (PD; PHYTRE PBR-LTS RX-30-S standard photonics detector) for linewidth measurement. The ESA (Anritsu MS2665C) is utilized to demonstrate the frequency response.

**Figure 2:**
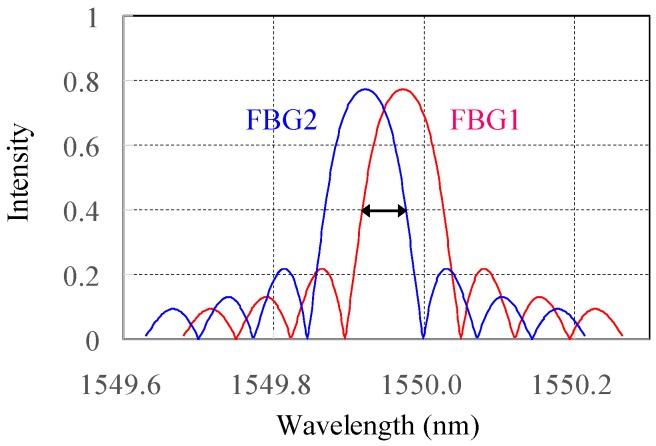
The two reflective FBG spectral correlation by FBG1 moving away from FBG2 during strain.

At the beginning, two FBGs with nearly the same reflective spectra are well aligned. The strain value ε of an axially loaded fiber is expressed as the sensing length change ratio to the original fiber length. When strain is applied onto the FBG1 through the stepper motor stage, the reflective FBG1 spectrum moves to the longer wavelength, relative to the FBG2 right spectral side, and the correlated area between the two FBG spectra becomes small, as shown in Figure 2. Since this kind of correlation linewidth on an OSA is not easily resolved due to its limited wavelength resolution of ~0.1 nm, the full width half-maximum (FWHM) of the optical field spectrum is measured using the interferometry-based linewidth characterization. The coherence or incoherence requirement should be carefully considered and satisfied.

In principle, an optical signal linewidth can be measured directly using an OSA. However, the finest conventional OSA resolution is in the order of 0.1 nm, which is approximately 12 GHz in a 1550 nm wavelength window and not suitable for a narrow linewidth characterization. In coherent detection, the input optical signal frequency is converted down to the RF domain by mixing it with a local oscillator. Self-homodyne detection eliminates the requirement for a local oscillator and the optical signal mixes with a delayed version of itself [16].

There are two typical linewidth characterizations—delayed self-homodyne and self-heterodyne. Both approaches are found to yield similar results, but the homodyne method is simpler to set up and gives a markedly better signal-to-noise ratio [17]. The delayed self-homodyne technique offers a simple approach to test an unmodulated optical source linewidth. Except for the presence of an optical frequency shifter in the delayed self-heterodyne case, the differential delay from two interferometers should exceed the coherence time of the optical source [18]. This spectral linewidth through cross-correlation is characterized using optical interferometers through low-loss fiber-optic delays. The optical circuits must deliver two fields to the photodetector, with and without being through the delayed optical path. 

**Figure 3:**
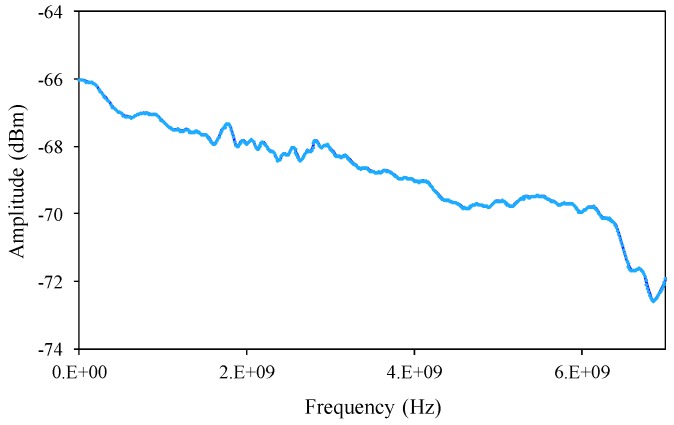
The characterized electrical spectrum response from ESA.

The linewidth information translation from the OSA to the electrical spectrum is illustrated in Figure 3. The characterized electrical spectrum response from ESA shows the original 4 GHz linewidth from two FBG spectral correlations without strain applied. The electrical spectrum response looks similar and its correlated linewidth decreases when the strain is applied to the FBG 1 from 0.8 to 6.4 με. The displayed electrical power spectrum has identical shapes to the actual optical spectrum. The reason for this is that the shape of these functions is preserved through the autocorrelation operation. The delayed self-homodyne mixing two FBGs’ cross-correlated spectra experiences the correlation after the delayed Mach–Zehnder interferometer. Only half of the symmetrical electrical spectrum needs to be considered because the mixing spectrum centers at 0 Hz [18].

## 4. Discussion

**Figure 4:**
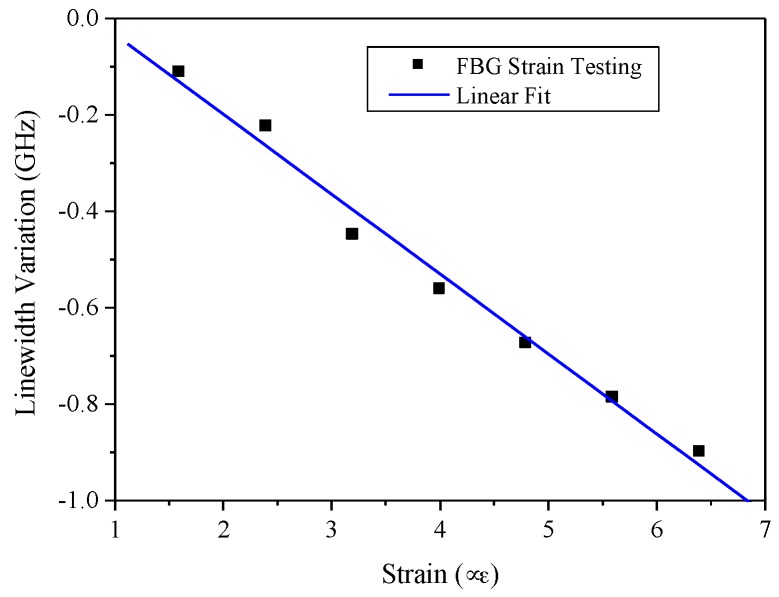
The fiber strain sensitivity from two FBG reflective spectrum correlation.

The linewidth information from the ESA through the delayed self-homodyne was analyzed through the implemented strain from 0.8 to 6.4 με. The linewidth decreased because the FBG1 reflective spectrum drifted out of the FBG2 spectrum window. The strain sensitivity for two FBG spectral correlations demonstrates 166 MHz/με with a negative slope and the linear regression, R, is 0.991, as shown in Figure 4. The two FBGs used for these strain experiments were overlapped for around 4 GHz in linewidth. The linewidth decreases when the stepper motor is applied on the FBG. Due to our motor stage limit, the smallest strain we could obtain is 0.8 με. To the best of our knowledge, the photo elastic effect cannot be easily implemented into the fiber Bragg grating for modal calculation. Therefore, we can only conclude that the linear range between the strain and spectral linewidth interrogation is from 0.8 to 6.4 με.

**Figure 5:**
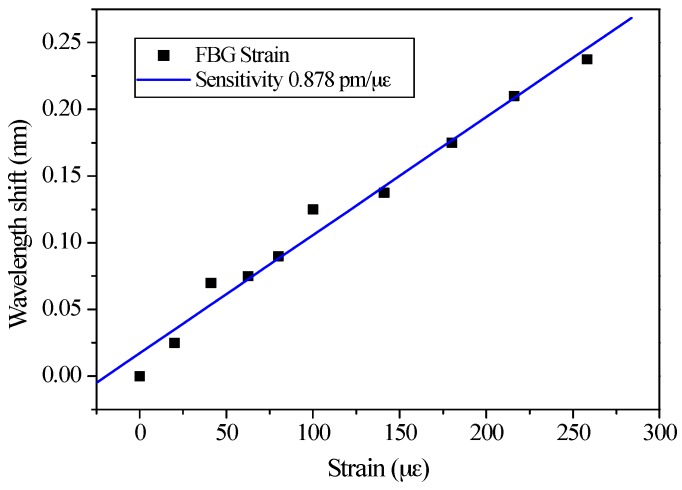
The fiber strain sensitivity from the FBG Bragg wavelength shifts.

For comparison, Bragg wavelengths were utilized for strain sensing characterization from 25 to 260 με, and the sensitivity was 0.878 pm/με, which means that 1 με requires an approximately 1 pm wavelength resolution in the OSA, as shown in Figure 5. Due to the limited wavelength resolution of 0.01 nm from OSA, the strain from 25 to 260 με is only applied for Bragg wavelength sensing because a strain lower than 25 με could not be detected in the OSA-based wavelength characterization.

To understand the photo elastic effect, the effective elastic-optic coefficient *γ* for the Bragg wavelength shift is −0.43 under a 1550 nm wavelength after inserting a 0.878 pm/με sensitivity into Equation (1). As for the two FBG spectral correlations, 166 MHz/με can be estimated as 1.33 pm/με at a 1550 nm operating wavelength. After the Bragg resonance wavelength shift sensitivity of 1.55 pm/με is considered from the previous GratingMOD calculation, the sensitivity difference of 0.22 pm/με can represent the FBG spectrum sensitivity broadening, which indicates a 0.44 pm/με sensitivity on two sides. We can conclude that the effective elastic-optic coefficient *γ* decreases the longer Bragg wavelength shift and broadens the FBG spectrum when a positive fiber strain is applied.

Since a two-FBG spectral linewidth moves with the cross-correlation wavelength under fiber strain, the Gaussian curve employed to model the correlated FBG spectrum wavelength with a random noise algorithm [6] for the highest strain resolution can be utilized to estimate the spectral linewidth resolution. In order to estimate the strain resolution, the assumptions are as follows:

The Gaussian function order can be taken as 1 and the ESA frequency resolution from the 5 GHz bandwidth is 0.01 GHz through the 500 points from the MS2665C data output port, in which the linewidth resolution and bandwidth approximately represent 0.08 pm and 0.04 nm, respectively, at a 1550 nm wavelength. The intensity-noise level for the photodetector can be −30 dB and the scanning frequency inaccuracy of MS2665C is around 1 pm at a 1550 nm wavelength. 

Compared with the typical wavelength resolution of 0.1 nm from OSA, the lowest frequency limit of kHz from ESA is much lower and therefore owns the potential for higher fiber strain sensing resolution. Therefore, the strain resolution is derived as 50 nε [6] and could be improved up to 10 nε when the two interrogated FBG spectral linewidth through correlation is reduced to 1 GHz.

## 5. Conclusions

The FBG is typically utilized to sense fiber strain using Bragg wavelength shifting. For better strain resolution, we successfully demonstrated fiber sensing technology through the correlation linewidth from two FBG reflective spectra. After the photo elastic effect was considered, one FBG spectral linewidth experienced broadening around 0.44 pm/με during strain. The strain sensitivity and resolution were 166 MHz/με and 50 nε, respectively, based on the ESA. The strain sensing resolution using two interrogated FBG reflective linewidth can be expected to yield values higher than 10 nε at bandwidths lower than 1 GHz through cross-correlation.
